# A Simple Method to Improve Autonomous GPS Positioning for Tractors

**DOI:** 10.3390/s110605630

**Published:** 2011-05-26

**Authors:** Jaime Gomez-Gil, Sergio Alonso-Garcia, Francisco Javier Gómez-Gil, Tim Stombaugh

**Affiliations:** 1 Department of Signal Theory, Communications and Telematics Engineering, University of Valladolid, 47011 Valladolid, Spain; E-Mail: salonsog@ribera.tel.uva.es; 2 Department of Electromechanical Engineering, University of Burgos, 09006 Burgos, Spain; E-Mail: fjggil@ubu.es; 3 Biosystems and Agricultural Engineering Department, University of Kentucky, Lexington, KY 40546, USA; E-Mail: tstomb@bae.uky.edu

**Keywords:** agricultural vehicles, control, Global Positioning System (GPS), guidance

## Abstract

Error is always present in the GPS guidance of a tractor along a desired trajectory. One way to reduce GPS guidance error is by improving the tractor positioning. The most commonly used ways to do this are either by employing more precise GPS receivers and differential corrections or by employing GPS together with some other local positioning systems such as electronic compasses or Inertial Navigation Systems (INS). However, both are complex and expensive solutions. In contrast, this article presents a simple and low cost method to improve tractor positioning when only a GPS receiver is used as the positioning sensor. The method is based on placing the GPS receiver ahead of the tractor, and on applying kinematic laws of tractor movement, or a geometric approximation, to obtain the midpoint position and orientation of the tractor rear axle more precisely. This precision improvement is produced by the fusion of the GPS data with tractor kinematic control laws. Our results reveal that the proposed method effectively reduces the guidance GPS error along a straight trajectory.

## 1. Introduction

Agricultural vehicle guidance has undergone impressive advances in recent years [1–6]. Autonomous guidance is mainly based on two techniques: positioning and control. Tractor positioning may be done globally and/or locally. Laser triangulation and Global Navigation Satellite Systems (GNSS) are global positioning systems used in tractor guidance. Machine vision, accelerometers, gyros, Inertial Navigations Systems (INS), electronic compasses, radars and other devices are all used as local positioning systems [1–4]. Tractor guidance can be achieved using either a local or global positioning system, or even by using several positioning systems simultaneously. Several positioning systems involve multiple sensors which in turn require sensor fusion techniques to combine the information they provide. The Kalman filter [7] is usually employed in this task. There are multiple examples of agricultural autonomous guidance in the scientific literature that only apply either Global Positioning System (GPS) positioning [8–12], machine vision positioning [13–16] or examples that use multiple positioning sensors [17–20].

Control laws for autonomous tractor guidance can be developed by modeling the tractor with a simple kinematic model [8,11,21]. Very accurate or high-speed guidance requires that certain effects such as inertia, sliding and springing be taken into account in the tractor model. Dynamic tractor models may be employed [22–24] for that purpose. Fuzzy logic [9,14] and neural network [17,25] controllers have also been used for autonomous tractor guidance.

One way to improve tractor GPS guidance performance is to employ a more precise tractor positioning system, which can be obtained by means of: (i) a better receiver, (ii) more effective differential GPS corrections or (iii) the fusion of GPS with other local positioning systems such as electronic compasses or Inertial Navigation Systems (INS). These solutions generally result in a more expensive system. This article presents, in contrast, a simple and low-cost method to improve GPS tractor positioning using only a GPS receiver as the positioning sensor. The method is based on placing the GPS receiver in a forward position where changes of the tractor’s orientation influence the GPS trajectory more, to obtain a more precise tractor positioning taking into account the data received from the GPS receiver and the tractor kinematic laws. Although researchers such as Stombaugh *et al.* [21] stated that the placement of a GPS receiver influences guidance performance, to the best of our knowledge there are no papers in the agricultural scientific literature that have explored this method.

## 2. Materials and Methods

### 2.1. Kinematic Model

**Figure 1.**
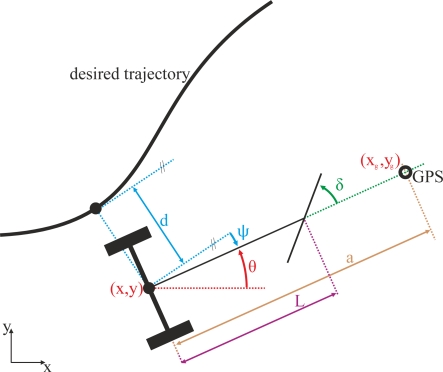
Tractor schematic and variables description.

A tractor has front-wheel steering and the rear wheels are forward-driven without being steered. The inputs to this system are therefore the driving speed, u, and the front wheel steering angle, δ. The tractor behavior can be described with a vector in the space state q, defined by the expression:

(1) $$\text{q}\;=\;{\left[\text{x},\;\text{y},\;\theta ,\;\delta \right]}^{\text{T}}$$

where x, y represent the position in Cartesian coordinates of the midpoint of the rear wheel axle, O, θ is the orientation of the vehicle with respect to the X-axis and δ is the steering angle of the front wheels with respect to the forward direction of the vehicle. L is the length from O to the center of the front axle. Figure 1 shows a schematic of the system and the variables description.

Assuming no-slip conditions on the wheels, the kinematic model of the vehicle is given by:

(2) $$\begin{array}{lll} \dot{\text{x}} & = & \text{u}\;\text{cos}\;\theta \\ \dot{\text{y}} & = & \text{u}\;\text{sin}\;\theta \\ \dot{\theta } & = & \frac{\text{u}}{\text{L}}\text{tan}\;\delta \end{array}$$

### 2.2. Position and Orientation Computation of the Rear Axle Midpoint from GPS Positions: Method 1

**Figure 2.**
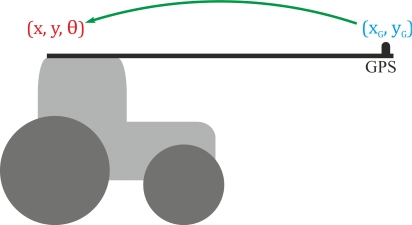
The position and orientation at the midpoint of the rear axle (x, y, θ) have to be computed from positions received from the GPS placed in a forward position (x_G_, y_G_).

The position of the rear axle midpoint and the orientation of the tractor have to be known so that guidance control may be performed more easily. In this article, this information is obtained from the positions supplied by the GPS receiver, which is placed in a forward position (Figure 2).

The tractor position (x, y, θ) at the midpoint rear wheel axle can be obtained from the positions obtained from the GPS receiver (x_g_, y_g_), either with the kinematic tractor model or from geometric relations.

The geometric relationship between (x, y, θ) and (x_g_, y_g_) is:

(3) $$\begin{array}{lll} {\text{x}}_{\text{g}} & = & \text{x}\;+\;\text{a}\;\text{cos}\;\theta \;\\ {\text{y}}_{\text{g}} & = & \text{y}\;+\;\text{a}\;\text{sin}\;\theta \end{array}$$

where a is the distance between the GPS receiver position and the rear axle midpoint of the tractor.

Differentiation with respect to time in Equation (3) and then by applying Equation (2):

(4) $${\dot{\text{x}}}_{\text{g}}\;=\;\dot{\text{x}}\;-\;\text{a}\;\dot{\theta }\;\text{sin}\;\theta \;=\;\text{u}\;\text{cos}\;\theta \;-\;\text{a}\;\dot{\theta }\;\text{sin}\;\theta$$

(5) $${\dot{\text{y}}}_{\text{g}}\;=\;\dot{\text{y}}\;+\;\text{a}\;\dot{\theta }\;\text{cos}\;\theta \;=\;\text{u}\;\text{sin}\;\theta \;+\;\text{a}\;\dot{\theta }\;\text{cos}\;\theta$$

From Equations (4) and (5):

(6) $$\begin{array}{c} {\left(4\right)}^{2}\;+\;{\left(5\right)}^{2}\;\Rightarrow \\ {\dot{\text{x}}}_{\text{g}}^{2}\;+\;{\dot{\text{y}}}_{\text{g}}^{2}\;=\;{\left(\text{u}\;\text{cos}\;\theta \;\;-\;\text{a}\dot{\theta }\;\text{sin}\;\theta \right)}^{2}\;+\;{\left(\text{u}\;\text{sin}\;\theta \;+\;\text{a}\dot{\theta }\;\text{cos}\;\theta \right)}^{2}\\ ={\text{u}}^{2}\;{\text{cos}}^{2}\;\theta \;+\;{\text{a}}^{2}\;{\dot{\theta }}^{2}\;{\text{sin}}^{2}\;\theta \;-\;2\;\text{u}\;\text{a}\;\dot{\theta }\;\text{cos}\;\theta \;\text{sin}\;\theta \\ +\;{\text{u}}^{2}\;{\text{sin}}^{2}\;\theta \;+\;{\text{a}}^{2}\;{\dot{\theta }}^{2}\;{\text{cos}}^{2}\;\theta \;+\;2\;\;\text{u}\;\text{a}\;\dot{\theta }\;\text{cos}\;\theta \;\text{sin}\;\theta \;=\;{\text{u}}^{2}\;+\;{\text{a}}^{2}{\dot{\theta }}^{2} \end{array}$$

and then, from Equation (6):

(7) $$\dot{\theta }\;=\;\pm \;\frac{1}{\text{a}}\;\sqrt{{\dot{\text{x}}}_{\text{g}}^{2}\;+\;{\dot{\text{y}}}_{\text{g}}^{2}\;-\;{\text{u}}^{2}}$$

Then, the position and orientation of the tractor can be obtained in a recursive way using the position obtained from the GPS in a forward position and the previous tractor state, as follows:

(8) $$\theta [\text{n}]\;=\;\theta [\text{n}\;-\;1]\;\pm \;\frac{\Delta \text{T}}{\text{a}}\;\sqrt{\left(\frac{{\text{x}}_{\text{g}}[\text{n}]\;-\;{\text{x}}_{\text{g}}{\left[\text{n}\;-\;1\right]}^{2}}{\Delta \text{T}}\right)\;+\;{\left(\frac{{\text{y}}_{\text{g}}[\text{n}]\;-\;{\text{y}}_{\text{g}}{\left[\text{n}\;-\;1\right]}^{2}}{\Delta \text{T}}\right)}^{2}\;-\;{\text{u}}^{2}\;}$$

(9) x[n] = x[n − 1] + u ΔT cos(θ [n])

(10) y[n] = y[n − 1] + u ΔT sin(θ [n])

Equations (8–10) must be sequentially computed at each new GPS position, where ΔT is the time between the reception of two GPS positions. Equation (8) offers two possibilities for θ[n], and thus two sets of (x[n], y[n], θ[n]) are obtained. With each one, (x_g_[n], y_p_[n]) can be geometrically computed, and any set that does not match the real position received by the GPS may be discarded.

### 2.3. Position and Orientation Computation of the Rear Axle Midpoint from GPS Positions: Method 2

**Figure 3.**
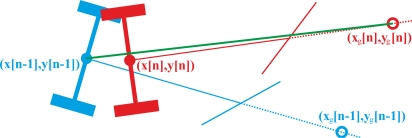
Two near positions of the tractor when following a curved trajectory.

The computational cost of the previous section can be reduced by an approximation, taking into account Figure 3.

Equation (11) is obtained if it is assumed that (x[n], y[n]) lies along the straight green line that joins (x[n − 1], y[n − 1]) with (x_g_[n], y_g_[n]) in Figure 3. Once θ is approximately calculated, x[n] and y[n] may be obtained with Equations (12) and (13):

(11) $$\theta [\text{n}]\;=\;\text{atan}\;\left(\frac{{\text{y}}_{\text{g}}[\text{n}]\;-\;\text{y}[\text{n}\;-\;1]}{{\text{x}}_{\text{g}}\;[\text{n}]\;\;-\;\text{x}[\text{n}\;\;-\;1]}\right)$$

(12) $$\text{x}[\text{n}]\;=\;{\text{x}}_{\text{g}}[\text{n}]\;-\;\text{a}\;\;\text{cos}(\theta \;[\text{n}])$$

(13) $$\text{y}[\text{n}]\;=\;{\text{y}}_{\text{g}}[\text{n}]\;-\;\text{a}\;\;\text{sin}(\theta \;[\text{n}])$$

Our experimental tests revealed that when (x[n], y[n]) is near to (x[n − 1], y[n − 1]) this approximation is accurate. This happens when the speed of the tractor is low and the GPS provides positions at 1 Hz, or when the GPS position rate is higher than 5 Hz at any usual tractor speed.

### 2.4. Control Law

The control law employed by Noguchi *et al.* [17], Stoll *et al.* [10] and Nagasaka *et al.* [20] among others, which is given by Equation (14), was chosen to test the system. It is one of simplest used in agricultural GPS guidance vehicles. The steering angle is proportional to the error in distance d, and the error in orientation Ψ:

(14) $$\delta \;=\;{\text{k}}_{1}\text{d}\;+\;{\text{k}}_{2}\;\psi$$

The following steps were taken to choose the control gains k_1_ and k_2_ for the experiments: (i) initially, k_1_ = 0 was fixed and a search was made for the maximum value of k_2_ that did not cause oscillations in the motion of the system. (ii) Subsequently, k_2_ was fixed as 70% of this maximum value. (iii) The selected value for k_2_ was used to find the maximum value of k_1_ before oscillation occurred in the motion of the tractor. Then, k_1_ was fixed as 80% of this maximum value. This tuning was done at a speed of 1 m/s. This experimental procedure is similar to the second Ziegler-Nichols experimental method for tuning PID controllers [26], because tractor orientation error ψ acts in a similar way to speed at which the distance to the trajectory increases or decreases, which is similar to the differential part of a PID control.

### 2.5. GPS Errors

GPS receivers produce errors. **Absolute errors** are the distance between real positions and positions obtained by the GPS. Tractors usually make passes along fields with a constant separation. The first pass is typically done manually by the tractor driver. With autonomous guidance systems, the next passes are done automatically by the guidance system with the desired separation between passes. With assisted guidance systems, the next passes are done manually but in an assisted way by means of the guidance system, at the desired separation between passes. In both situations, **relative errors** occur, which are the distance differences between the desired separations between passes and the real separations between passes.

Relative errors are smaller than absolute errors. In agricultural tasks, farmers notice relative error, which has led companies such as John Deere or Trimble to provide information on relative errors in their GPS-guidance-systems specifications that they refer to as “pass-to-pass accuracy”. Relative errors in tractor GPS guidance systems are usually subject to some conditions, such as Dilution of Precision (DOP) and time between passes. The common time between passes in data sheet specifications is 15 minutes, because this is the habitual time between tractor passes in medium-sized plots.

**Figure 4.**
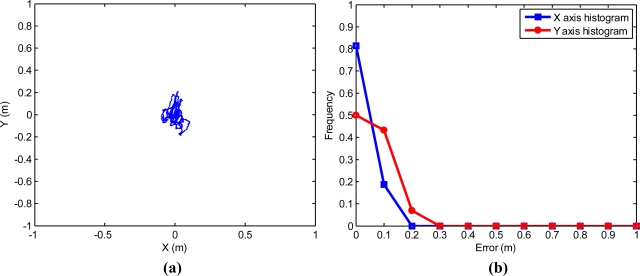
**(a)** 900 UTM positions obtained by a static Haicom HI-204III. **(b)** Histogram of these errors.

Figure 4(a) shows 900 Universal Transverse Mercator (UTM) positions offered by a stationary Haicom HI-204III over a 15-minute time frame. These 900 UTM positions are centered over their UTM mean. The GPS used to take these positions had the static navigation disabled and provided one position per second, and in this way, 900 points were obtained in 15 minutes. Figure 4(b) shows the histograms of relative errors along the X and Y axes when the real value is considered as the mean of all positions. It can be observed that the relative error of this low cost GPS stays below 40 cm on measures over 15 minutes. The standard deviation was 10.7 cm in X axis, and 11.9 cm in Y axis.

The relative errors in these 900 points are used in the section Simulation Results with Experimental GPS Errors.

Taking the previous data into account, a low cost Haicom HI-204III GPS receiver can technically be used to guide a tractor when the time between passes is short and precision is not required. The distance between passes could differ by large amounts from the desired when a long time between passes occurs.

### 2.6. Experimental System

**Figure 5.**
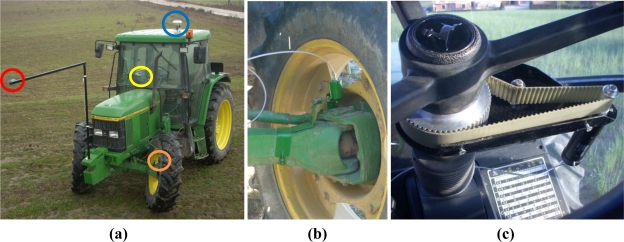
**(a)** 6400 John Deere tractor used in the tests. The red circle indicates the position of the Haicom HI-204III navigation GPS, the blue circle the position of the Trimble R4 reference GPS, the orange circle the position of the magnetic encoder, and the yellow circle the position of the DC motor and pulley. **(b)** Magnetic encoder used for measuring the steering angle. **(c)** DC motor and pulley used for moving the steering system.

A 6400 John Deere tractor was equipped with devices to perform autonomous tractor guidance (Figure 5). A DC RE-30 Maxon motor was used to move the steering wheel and a reducer gear was employed to adapt motor speed to steering wheel speed. A striated pulley connected the reducer gear to the steering wheel. The reduction rate was 14:1 for the reducer gear and 6:1 for the pulley. A magnetic encoder was used to measure steering position and a microcontroller was used to control the steering system. A low cost Haicom HI 204III GPS receiver configured to process the European Geostationary Navigation Overlay Service (EGNOS) corrections was used as the navigation GPS. This is a C/A code GPS receiver that provides a 1 Hz position rate, integrates a Sirf Start III chipset and offers an estimated accuracy of 5 m (2D RMS) with EGNOS augmentation. An R4 Trimble receiver was used to measure with precision the trajectories followed by the tractor. This is a high end dual frequency receiver, which when working with carrier-phase measurements in Real Time Kinematic (RTK) mode, offers up to 1 cm accuracy. RTK corrections were provided through a General Packet Radio Service (GPRS) cellular communication from a Virtual Reference Station (VRS) managed by ITACyL, a Spanish regional agrarian institute. The VRS augmentation technology usually allows centimeter-level accuracy to dual frequency receivers when working in RTK mode [27].

**Figure 6.**
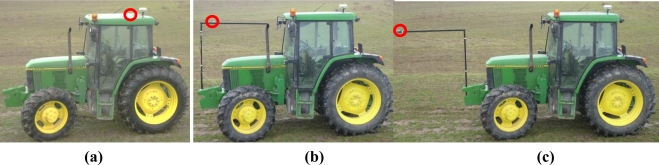
Different positions of the Haicom HI-204III navigation GPS in the field tests. The navigation sensor position is highlighted with a red circle. **(a)** The guidance GPS placed in the back part of the cabin over the real axle. **(b)** The guidance GPS placed 3 m ahead of the rear axle of the tractor. **(c)** The guidance GPS placed 5 m ahead of the rear axle of the tractor.

Figure 6 shows three placements of the navigation GPS in the field tests at 0, 3 and 5 meters ahead of the tractor rear axle. A low height placement of the navigation GPS would be possible in forward positions, and this would reduce the errors due to rough test surface. But, in order to compare only the distance from the rear axle, the placement height was always the same.

### 2.7. Methods

**Figure 7.**
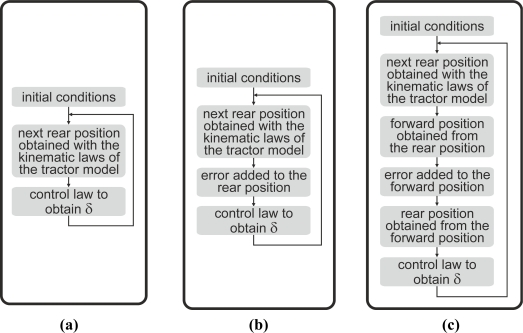
Flow charts of the procedures to simulate **(a)** without errors, **(b)** with errors added in rear position and **(c)** with errors added in a forward position.

The methodology of this article comprises certain simulations and field tests to prove the performance of the proposed system. Some simulations of autonomous tractor guidance along a straight line and a step response, according to Figure 7, were computed without errors [Figure 7(a)], adding errors at the rear axle position [Figure 7(b)] and adding errors at forward positions [Figure 7(c)].

In these simulations, the kinematic model of the tractor presented in Equation (2) and the control law of Equation (14) were employed. The simulation rate was 1 Hz. Constants L = 2.3 m, u = 1 m/s, and the control gains obtained experimentally in the field test (k_1_ = 0.08, k_2_ = 0.5) were fixed. Experimental errors of Figure 4 were added to each analytically calculated position of the tractor, according to the flow chart in Figure 7. These added errors were experimentally obtained with the same Haicom HI 204III GPS receiver used as the guidance GPS in the field tests. These errors were graphically shown in Figure 4(a). For each simulation, the difference between two consecutive data positions obtained from the receiver fixed at a static point, was added as the relative error.

Additionally, field tests were performed with the system described in the experimental system section. The guidance along a straight path, the step response impulse and the maximum stable speed were tested.

## 3. Results and Discussion

### 3.1. Simulation Results with Experimental GPS Errors

**Figure 8.**
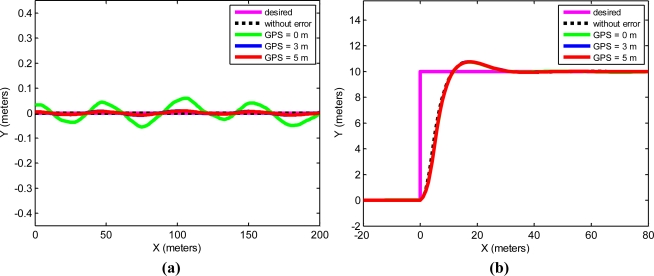
**(a)** Simulation results obtained when the tractor follows a straight line and experimental errors are added. **(b)** Simulation results obtained in a step response trajectory when experimental errors are added.

Simulations of autonomous tractor guidance along a straight line and a step response were performed on a rough plot. The initial conditions for the tractor were (x, y, ψ, δ) = (0, 0, 0, 0), and the desired trajectory in the step response was y = 2 m. Figure 8(a,b) were obtained from the straight line and step response tracking.

Simulations with different forward GPS guidance positions were performed in order to compute the mean and Root Mean Square (RMS) values of the error. Simulation time was 14 minutes (14 × 60 = 840 points) and the reference trajectory was a straight line. Table 1 shows the obtained results.

### 3.2. Experimental Results

Three kinds of tests were performed with the GPS placed ahead of the rear axle at three different distances: the first found the error in the continuous tracking of a straight trajectory; the second tested the step response behavior; the third measured the maximum speed that keeps the guidance control. The control law used for the tests is given by Equation (14). The control law parameters were fixed at k_1_ = 0.08 and k_2_ = 0.5, values experimentally obtained at 1 m/s speed, following the tuning procedure described in the methods section.

**Figure 9.**
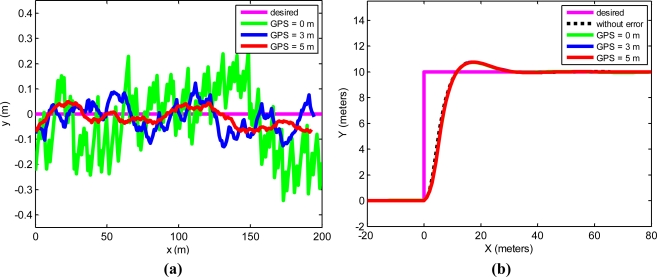
**(a)** Distance error from a desired straight trajectory at 1 m/s with the GPS placed at 0, 3 and 5 meters from the rear axle of the tractor. **(b)** Step response tracking at 1 m/s with the GPS receiver placed at 0, 3 and 5 meters from the rear axle of the tractor.

Figure 9(a) shows the trajectory distance error of the straight trajectory. In this figure, it is possible to observe that the error is reduced when the GPS receiver is placed ahead of the tractor. Figure 9(b) shows the trajectories in the tracking of a step response with the GPS placed at 0, 3 and 5 m from the rear axle of the tractor. No major differences were observed regarding the GPS receiver position in the step response.

It is worth mentioning that it was necessary to shift some of the trajectories on the X and Y axes in Figure 9(a,b), due to the behavior of the low-cost Haicom HI-204III guidance GPS receiver that was employed. It offers good precision or relative accuracy in the short periods of time necessary to perform a specific trajectory test, but poor absolute accuracy between the longer periods of time that two specific trajectory tests entail. Buick [28] provides more detailed information on the meaning of the terms relative accuracy or precision and absolute accuracy or accuracy.

The standard deviation of the field test was also computed when the tractor followed the straight line trajectory and the results are shown in Table 2.

The Haicom HI-204III guidance GPS receiver offers a rate of position data of 1 Hz. With this low rate, only low speed autonomous guidance is possible. In the third field test, the desired trajectory was a straight line. From an initial value of 1 m/s, the tractor speed was gradually increased. It was observed that the guidance error was increasing as the speeds increased. Moreover, the maximum speed at which the system remained stable was measured for each GPS position. These values are shown in Table 3.

### 3.3. Discussion

**Figure 10.**
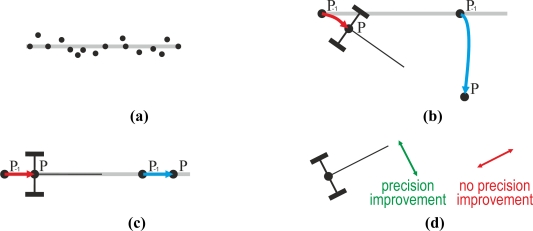
**(a)** The straight line represents a trajectory followed by a tractor and the dots represent the positions provided by a GPS receiver placed on the tractor. **(b)** Trajectory of the rear axle of a tractor that turns (red) and trajectory of a GPS placed ahead of the same tractor (blue). **(c)** Trajectory of the rear axle of a tractor that goes straight (red) and trajectory of a GPS placed ahead of the same tractor (blue). **(d)** Directions with respect to the tractor orientation where the proposed method improves the positioning (green) or does not improve the positioning (red).

Results show that the proposed method improves the guidance performance. This improvement can be intuitively understood. Figure 10(a) sketches the positions that a GPS receiver provides when a tractor goes along a straight line. These positions are not on the straight line due mainly to: (i) the error of the GPS receiver and to (ii) the oscillations that the tractor suffers when it goes along the common rough farm terrain. Figure 10(b) sketches the trajectories of the rear axle and of a GPS receiver placed on a tractor that turns. The red line represents the trajectory of the rear axle of this tractor. The blue represents the trajectory acquired by the GPS placed ahead of the tractor. It can be clearly observed in this figure that the blue trajectory is longer than the red. Our proposed method computes the red trajectory of the tractor rear axle with the blue trajectory acquired by the GPS receiver placed ahead of the tractor. In this process, the rear axle positioning error decreases at a ratio similar to the length of the blue trajectory divided by the length of the red trajectory. It is interesting to realize that lengths of the blue and red trajectories are the same when the tractor goes straight [Figure 10(c)]. Figure 10(b,c) suggest that: (i) the proposed method improves the tractor orientation positioning information and that (ii) the proposed method improves the positioning precision in the transverse axis of the tractor direction, but does not improve the positioning precision over the tractor direction, as Figure 10(d) illustrates.

A more formal explanation of this precision improvement is that more information for the positioning is used in the proposed system; therefore, the tractor positioning is more precise. The data GPS receiver is only used to identify the position of the tractor when the GPS receiver is placed on the tractor rear axle. But with the GPS in a forward position, the rear position of the tractor is computed with the positions of the GPS receiver together with the kinematic model of the tractor laws. GPS data and kinematic model tractor laws are fused in the proposed method to position the tractor.

The proposed method employs only the GPS receiver to provide tractor positioning data. There was a steering angle encoder on the tractor. However, data from this sensor was only used by the steering system controller to help it achieve the desire steering angle.

The initial orientation of the tractor was provided to the system in our simulations and field tests, but an arbitrary value can be employed in the proposed method when the initial orientation of the tractor is not known. In this case, the proposed method would work poorly at the beginning of the trajectory, but after approximately one hundred iterations, it would work fine.

The Kalman filter can be used in GPS tractor guidance. This filter can be used to process (i) only the GPS receiver data or (ii) multiple sensors’ data. When it is used to process only the GPS receiver data, this filter produces an effect of smoothing [29]. When it processes the GPS data together with additional sensors’ data, this filter improves the tractor GPS positioning precision [20]. Then, Kalman filters can improve the tractor positioning in the same way as our proposed system. But Kalman filters require additional sensors and the system hardware would be further complicated.

Simulations and field test results of this article concur with the previous intuitive discussion. Concretely:

- The guidance errors in both Figure 8(a) and Figure 9(a) are reduced when the GPS is placed in a forward position. In our field tests, these errors are greater than in the simulations. This is because some error components are not modeled in simulations, for example, the positioning error introduced by the GPS receiver due to the tractor cab oscillations produced by the rough land used for the tests, typical of agricultural terrain.
- Differences were neither observed in the step response trajectories, nor in the simulations, nor in the field tests. This makes good sense, because when the tractor reaches the step, the distance to the desired trajectory is a large value of around 10 meters. The control law will give the maximum value in the δ angle with values close to 10 meters. A more precise positioning will not therefore produce a different response when the step happens.
- A cause that usually makes a real control system unstable is its noise level. From the data in Table 3, it may be deduced that the level of noise with the proposed system decreases.

This article, then, proves that by placing the GPS ahead of the tractor, it is possible to obtain more precise tractor positioning, and subsequently improve the autonomous GPS guidance performance of a guidance system that uses only a GPS receiver as the positioning sensor. This more precise tractor positioning is achieved because the proposed method uses the received GPS data and the kinematic model of the tractor together for tractor positioning, whereas positioning is only obtained with the received GPS data when the receiver is placed on the rear axle. Our results are in agreement with Stombaugh *et al.* [21], who stated that the placement of a GPS receiver influences guidance performance.

Some limitations should be considered. The proposed method has been tested in a guidance system that uses only a GPS receiver as the positioning sensor. Tractor guidance systems sometimes employ more positioning sensors, such as: (i) tilt and (ii) orientation sensors. When a tilt sensor is employed together with the GPS receiver, it is expected that the proposed method will also improve the positioning, but with a lower ratio. This is because in this situation the proposed method reduces only the GPS positioning error. It does not influence the positioning error caused by the oscillations that the tractor suffers when it goes through common rough farm terrain, because this error is eliminated by the tilt sensor. When a digital compass, a gyroscope, an INS or whatever other orientation sensor is employed together with the GPS receiver, it is expected that the proposed system will not improve the tractor GPS positioning. This is because the proposed system improves the precision of the orientation data obtained by the GPS positioning, but it is expected that these data were much more precisely provided by the orientation sensor.

## 5. Conclusions

By placing the GPS ahead of the tractor, as proposed in this article, it is possible to devise a simple and low cost method a more precise tractor positioning, and thus improve the autonomous GPS guidance performance. This more precise tractor positioning is achieved because the proposed method uses the received GPS data and the kinematic model of the tractor together for tractor positioning, whereas positioning is only obtained with the received GPS data when the receiver is placed on the rear axle. The proposed method improves tractor positioning when only a GPS receiver is used as the positioning sensor. The improvement in the positioning occurs in the tractor orientation and in the positioning with respect to the transverse axis of the tractor’s orientation. It is expected that the improvement ratio will decrease when a tilt sensor is employed and that no improvement will be achieved when the tractor positioning system includes an orientation sensor.

## Figures and Tables

**Table 1.** Mean and RMS errors obtained in the simulations with different forward GPS guidance positions.

	**Without added error**	**0 m**	**1 m**	**2 m**	**3 m**	**4 m**	**5 m**	**10 m**

**Mean error (cm)**	0	2.6	0.46	0.45	0.43	0.41	0.39	0.29
**RMS error (cm)**	0	3.0	0.53	0.51	0.49	0.47	0.45	0.34

**Table 2.** Standard deviation errors obtained in the field tests with different forward GPS guidance positions.

	**0 m**	**3 m**	**5 m**
**Standard deviation (cm)**	16	4.7	3.7
**RMS error (cm)**	32	5.7	4.8

**Table 3.** For each advance from the rear axle, this table presents the maximum speed of the tractor that kept the system stable.

**GPS ahead of the rear axle**	**0 m**	**3 m**	**5 m**
**Speed that leads to guidance instability**	1.5 m/s	2.4 m/s	2.6 m/s
